# Traffic Jams in the Brain: How Kinesin Dysfunction Shapes Neurodevelopmental Disorders

**DOI:** 10.3390/cimb48080837

**Published:** 2026-08-18

**Authors:** Mohammad Sadegh Shams Nosrati, Morteza Doustmohammadi, Alireza Dostmohammadi, Armita Kakavand Hamidi, Mahsa Boogari, Zahra Hoseini Tavassol, Shakiba Khosravinejat, Majid Asgari, Morvarid Shafiei, Amir Hesam Nemati, Ferruccio Romano, Valeria Capra, Bruno Sterlini, Mohammad Darbalaei, Mohammad Salehi, Mir Davood Omrani, Federico Zara, Zoha Kibar, Tatsuo Miyamoto, Marcello Scala

**Affiliations:** 1 Medical Genetics Unit, IRCCS Istituto Giannina Gaslini, 16147 Genoa, Italy; 2 Department of Neuroscience, Rehabilitation, Ophthalmology, Genetics, Maternal and Child Health (DINOGMI), University of Genoa, 16132 Genoa, Italy; 3 Department of Molecular Virology, Pasteur Institute of Iran, Tehran P.O. Box 13169-43551, Iran; 4 Metabolic Disorders Research Center, Endocrinology and Metabolism Molecular-Cellular Sciences Institute, Tehran University of Medical Sciences, Tehran P.O. Box 14117-13135, Iran; 5 Department of Bioinformatics and Computational Biophysics, Faculty of Biology and Centre for Medical Biotechnology (ZMB), University of Duisburg-Essen, 45141 Essen, Germany; 6 Department of Medical Genetics, School of Medicine, Tehran University of Medical Sciences, Tehran P.O. Box 14618-84513, Iran; 7 Endocrinology and Metabolism Research Center, Endocrinology and Metabolism Clinical Sciences Institute, Tehran University of Medical Sciences, Tehran P.O. Box 14117-13135, Iran; 8 Research Center, Alborz University of Medical Sciences, Karaj 3198764653, Iran; 9 Myeloma Center, Winthrop P. Rockefeller Institute, Department of Internal Medicine, University of Arkansas for Medical Science, Little Rock, AR 72205, USA; 10 Department of Bacteriology, Pasteur Institute of Iran, Tehran P.O. Box 13169-43551, Iran; 11 Department of Epidemiology and Biostatistics, Pasteur Institute of Iran, Tehran P.O. Box 13169-43551, Iran; 12 Genomic and Clinical Genetics Unit, IRCCS Istituto G. Gaslini, 16147 Genoa, Italy; 13 Dipartimento di Medicina Sperimentale, Università di Genova, 16132 Genoa, Italy; 14 IRCCS Azienda Ospedaliera Metropolitana, 16132 Genoa, Italy; 15 West German Cancer Center, Department of Medical Oncology, University Hospital Essen, University of Duisburg-Essen, 45147 Essen, Germany; 16 Department of Medical Genetics, Faculty of Medicine, Shahid Beheshti University of Medical Sciences, Tehran P.O. Box 1985717443, Iran; 17 Azrieli Research Center of CHU Sainte Justine, University of Montreal, Montreal, QC H3T 1C5, Canada; 18 Division of Advanced Genome Editing Therapy Research, Research Institute for Cell Design Medical Science, Yamaguchi University, Ube 755-8503, Japan; 19 Department of Molecular and Cellular Physiology, Graduate School of Medicine, Yamaguchi University, Ube 755-8505, Japan

**Keywords:** kinesins, KIF motor proteins, axonal transport, microtubules, neurodevelopmental disorders, kinesinopathies, *KIF1A*, *KIF5A*, neuronal migration, precision medicine

## Abstract

The development and maintenance of the nervous system depend on a tightly regulated intracellular transport network in which kinesin superfamily (KIF) motor proteins drive microtubule-based delivery of synaptic vesicle precursors, organelles, mRNAs, and signaling components along axons and dendrites. Disruption of this machinery underlies a clinically heterogeneous spectrum of neurodevelopmental disorders (NDDs), including intellectual disability, epilepsy, autism spectrum disorder, microcephaly, malformations of cortical development, spasticity, and axonal neuropathy. Here, we synthesize current knowledge on how kinesin dysfunction shapes neurodevelopment. We outline the physiological roles of kinesins in neuronal polarity, organelle and mitochondrial positioning, synaptogenesis, and progenitor division, and survey principal disease-associated genes, including *KIF1A*, *KIF5A*, *KIF7*, *KIF11*, *KIF2A*, *KIF5C*, and emerging members such as *KIF14*, *KIF15*, and *KIF16B*. We detail how distinct pathogenic mechanisms, such as loss of motility, impaired cargo coupling, motor hyperactivity, mitotic spindle defects, and disrupted ciliary signaling, converge on shared cellular endpoints, and how tubulin isotypes and posttranslational modifications further modulate motor output. In this review, we discuss translational implications, including variant-resolved diagnosis and precision strategies to restore transport, dampen pathological hyperactivity, or stabilize the microtubule track. Collectively, these advances reframe kinesinopathies as mechanistically stratified disorders of neuronal transport.

## 1. Introduction

The spectacular complexity of the human nervous system, from initial cortical layering to the maintenance of meter-long axons, is fundamentally dependent on a massive, highly regulated intracellular logistics network. At the core of this system are the microtubule (MT) tracks and the kinesin superfamily (KIFs) of molecular motor proteins that power anterograde axonal transport [1]. During neurodevelopment, kinesins are essential for anterograde trafficking of synaptic vesicle precursors (*KIF1A*), mRNAs (via APC-kinesin-1 complexes), mitochondria (via Miro/TRAK-kinesin-1), and signaling receptors (e.g., TrkB, NMDAR subunits), thereby enabling neuronal migration, axon pathfinding, dendritic arborization, and synaptogenesis with spatiotemporal precision [2,3,4,5,6,7]. Disruptions in this delivery service, the literal “traffic jams in the brain”, are a major pathological mechanism underlying a diverse spectrum of debilitating neurodevelopmental disorders (NDDs), characterized clinically by intellectual disability, congenital brain malformations, spasticity, and neuropathy [8,9].

The kinesin superfamily, divided into multiple families, presents a paradigm of functional diversification, where distinct motor domains, stalk regions, and cargo-binding tails dictate the motor’s function and the resulting clinical phenotype upon mutation. Classical kinesinopathies, such as those caused by variants in *KIF5A* (Kinesin-1) and *KIF1A* (Kinesin-3), vividly illustrate the consequences of transport failure. For *KIF5A*-associated disorders, a clear genotype–phenotype correlation exists. Mutations in the N-terminal motor domain typically result in complicated Hereditary Spastic Paraplegia 10 (SPG10), while distinct variants in the C-terminal cargo-binding domain are linked to susceptibility to Amyotrophic Lateral Sclerosis (ALS25) [10,11]. However, the pathogenesis extends beyond simple motor disruption, encompassing defects in regulation and non-transport roles.

Kinesin activity is exquisitely controlled by autoinhibition, phosphorylation, and adaptor proteins to prevent runaway motility [12,13,14]. Pathogenic kinesin variants disrupt motor regulation through both loss- and gain-of-function mechanisms. Some reduce microtubule-stimulated ATPase and/or processive motility, as exemplified by *KIF1A* p.(R169T), which has been associated with severe NESCAV syndrome [15]. In contrast, variants such as p.(Val8Met), p.(Ala255Val), and p.(Arg350Gly) have been reported to increase *KIF1A* motor activity and are associated with hereditary spastic paraplegia phenotypes [16]. These observations suggest that variant-specific effects on motor behavior can contribute to differences in clinical presentation, although substantial phenotypic overlap and variable expressivity remain. Furthermore, the role of kinesins extends to non-canonical functions vital for development. The non-motor kinesin KIF26A, an unconventional member that lacks ATPase activity, is a powerful example, with biallelic loss-of-function variants responsible for Cortical Dysplasia, Complex, with Other Brain Malformations 11 (CDCBM11) [17,18]. This phenotype stems not from active transport failure, but from KIF26A’s critical roles in developmental signaling; KIF26A regulates GDNF-Ret signaling in enteric neuronal development [19], whereas KIF26A loss in the developing brain disrupts MAPK-associated programs, radial migration, axonal growth, and neuronal survival [17]. Similarly, the ciliary kinesin *KIF7* controls cortical development and connectivity through its regulation of the Sonic Hedgehog (SHH) signaling pathway, underscoring the tight overlap between transport, ciliopathies, and severe brain malformations (e.g., Acrocallosal Syndrome) [20,21]. Finally, the dosage and balance of the kinesin complex is vital, as demonstrated by the light chain component KLC2, where gain-of-function due to overexpression leads to Spastic Paraplegia, Optic Atrophy, and Neuropathy (SPOAN), showing that an imbalance in the complex can destabilize axonal architecture [22].

**Figure 1:**
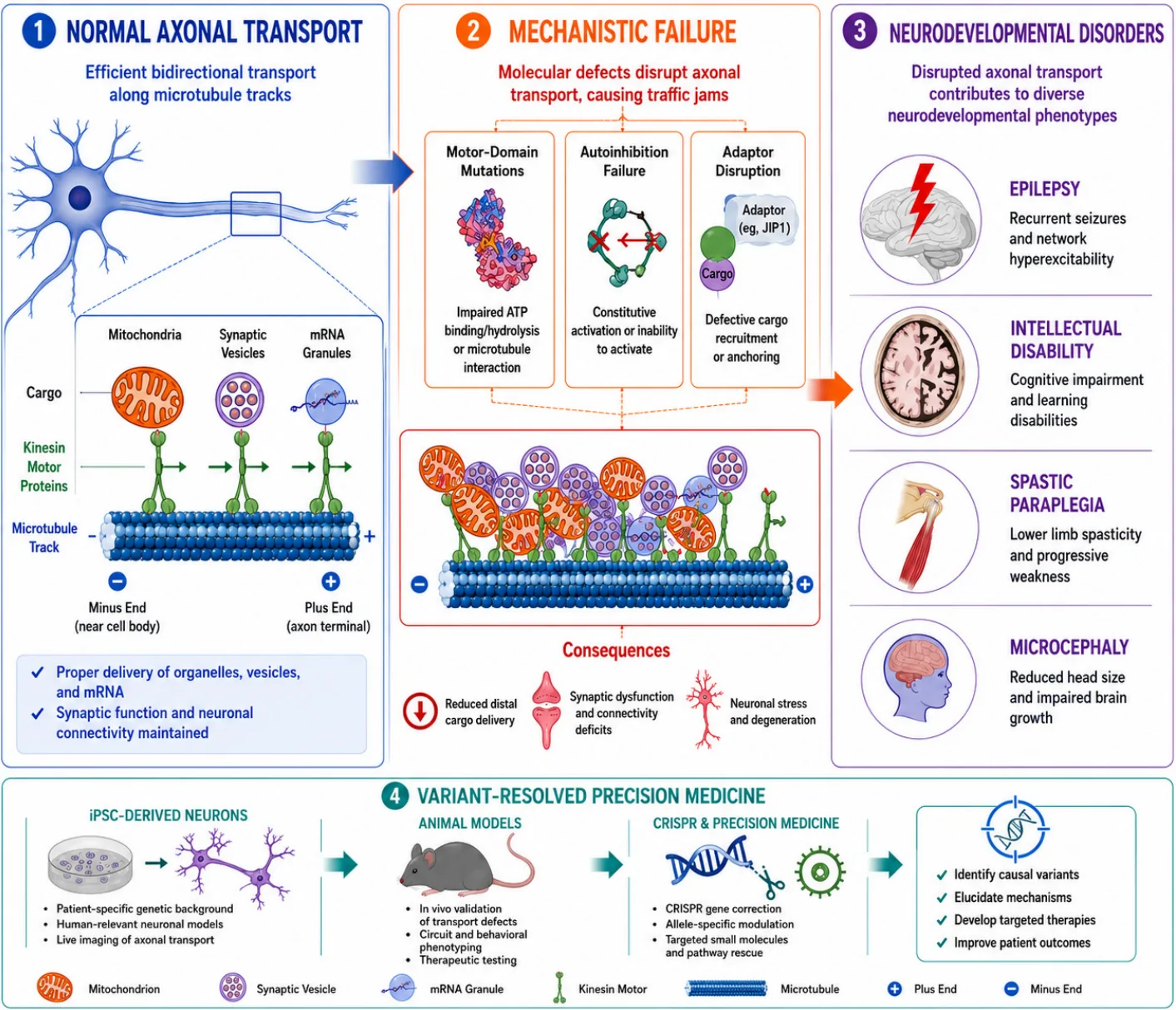
**Mechanistic framework of kinesin-mediated intracellular transport and its link to neurodevelopmental pathology.** The schematic illustrates the transition from normal cellular physiology to disease state. Normal neuronal function relies on kinesin-mediated transport of cargo (e.g., mitochondria, synaptic vesicles, mRNA) along microtubule tracks (**left**). Dysfunction in these motors, due to motor-domain mutations, autoinhibition failure, or adaptor disruption, leads to intracellular traffic jams and impaired neuronal development (**center**). This mechanistic breakdown correlates with a wide spectrum of clinical outcomes, including intellectual disability, epilepsy, and neurodegeneration (**right**), emphasizing the role of kinesinopathies in neurodevelopmental disease.

This review, which explores how kinesin dysfunction shapes neurodevelopmental disorders (NDDs), integrates foundational studies with the latest mechanistic insights. In this work, we provide a broader synthesis of more than 25 kinesin genes and regulatory proteins and organize them within an explicit mechanistic framework encompassing transport dysfunction, motor hyperactivity, cargo-coupling defects, mitotic and ciliary abnormalities, and alterations of the microtubule track. We will first synthesize the spectrum of NDDs associated with the kinesin superfamily, detailing the distinct pathologies arising from canonical versus atypical kinesin members. We will then examine the molecular mechanisms underlying kinesin dysfunction, including the consequences of motor-domain variants, alterations in cargo-binding regions, and disruption of regulatory adaptor proteins. A dedicated section on experimental model systems further connects these mechanisms with disease modeling and translational interpretation. Finally, we will discuss emerging therapeutic strategies aimed at restoring the functional balance of kinesin-mediated transport and consider their potential implications for precision medicine (Figure 1).

## 2. Kinesins in Brain Development

Neurons are highly specialized, polarized cells with two structurally and functionally distinct compartments. Axons transmit action potentials, whereas dendrites receive synaptic inputs [23]. Maintaining this polarity requires efficient long-range intracellular transport systems that deliver organelles, proteins, lipids, and macromolecular complexes to distal regions of the cell [24]. This transport is closely linked to the organization of microtubule networks, which provide tracks for motor proteins and help define compartment identity [25]. In addition, the axon initial segment (AIS) functions as a critical regulatory barrier that selectively filters cargo, preventing somatodendritic components from entering the axon and thereby preserving the fidelity of polarized transport and neuronal function [26,27].

The establishment and maintenance of neuronal polarity depend largely on differences in cytoskeletal architecture and motor-driven trafficking [28]. In axons, microtubules are arranged in a uniform plus-end-out orientation, favoring kinesin-mediated anterograde transport toward distal axonal regions. In contrast, dendrites display mixed microtubule polarity, enabling more complex and bidirectional transport patterns [29]. These structural differences are complemented by selective motor–cargo interactions that ensure precise targeting of proteins and organelles [30]. As neurons mature, dendritic microtubules undergo reorganization and stabilization, supporting functional specialization [25]. Collectively, these coordinated mechanisms ensure proper distribution of cellular components, which is essential for neuronal morphology, connectivity, and signaling capacity [29,31,32].

Kinesin motor proteins, particularly kinesin-1 (KIF5), play a central role in long-distance axonal transport [33]. KIF5 is a heterotetrameric motor complex composed of heavy and light chains, with the heavy chains containing motor domains and cargo-binding regions [13,23]. Its activity is tightly regulated through autoinhibitory and activation mechanisms that control cargo engagement and motility [13]. Functional studies have shown that loss of *KIF5A* reduces neurite complexity and impairs mitochondrial motility, highlighting its importance in neuronal development [34]. Transport selectivity is further refined at the proximal axon and AIS, where KIF5-associated cargos are preferentially allowed entry while somatodendritic cargos are excluded [26,27]. One of the most important cargos transported by KIF5 is mitochondria, which provide ATP and calcium buffering at distal neuronal sites [24]. Mitochondrial transport is mediated by adaptor proteins such as Miro and Milton/TRAK, which link mitochondria to kinesin motors [4]. Additionally, neuronal activity regulates mitochondrial positioning by detaching KIF5 from these complexes, leading to the immobilization of mitochondria at regions of high metabolic demand.

Intracellular transport is also critical for neuronal migration and early circuit formation, where precise spatial and temporal control of signaling molecules is required [35,36]. Endosomes function as key regulators in this process, acting as sorting hubs and signaling platforms [37,38]. Rab5-positive early endosomes regulate receptor internalization and early trafficking steps, and Rab5-dependent endosomal signaling contributes to neuronal dendritic development [35]. Rab11-dependent recycling pathways return receptors and membrane components to the cell surface, supporting neurite extension and cytoskeletal remodeling [36,39]. These pathways also interact with actin and microtubule networks to regulate neuronal morphology and outgrowth [36,39].

Rab GTPases act as nucleotide-dependent molecular switches that regulate membrane identity and motor recruitment. In their active GTP-bound state, Rabs recruit specific effector proteins that connect endosomal membranes to kinesin motors, either directly or through adaptor complexes, thereby coupling cargo identity and trafficking stage to directional microtubule-based transport. For example, Rab11 interacts with the effector Rab11-FIP5/Rip11 in a complex with kinesin-II to promote recycling endosome transport and receptor recycling [40,41]. The relevance of Rab-related trafficking pathways to neurodevelopmental disorders is illustrated by de novo variants in *DENND5B*, which encodes a DENN-domain Rab guanine nucleotide exchange factor and is associated with neurodevelopmental impairment, behavioral abnormalities, variable epilepsy, white matter abnormalities, and cortical gyration defects [42].

In parallel, neurons transport mRNAs and RNA-binding proteins into neurites, enabling localized protein synthesis that supports cytoskeletal dynamics and synapse formation [5,43]. Emerging evidence suggests that endosomal compartments also contribute to organizing mRNA localization and translation, further integrating trafficking with spatial proteome regulation [5,44].

Synapse development and plasticity are highly dependent on intracellular transport processes that ensure proper delivery of synaptic components [45]. During glutamatergic synaptogenesis, newly synthesized NMDA receptors assemble in the ER and are exported to the Golgi after proper NR1-NR2 assembly masks ER retention signals [46], before being transported into dendrites in microtubule-dependent transport packets [47]. Two major models have been proposed for NMDA receptor recruitment to nascent synapses: direct vesicular delivery and insertion at synaptic sites following axon-dendrite contact [47,48], or insertion into the dendritic plasma membrane followed by lateral diffusion to sites of synaptic contact [49,50]. KIF17 mediates dendritic transport of GluN2B/NR2B-containing vesicles via adaptor complexes involving Mint1 and CASK/Veli. Live imaging shows that KIF17-positive vesicles move processively along dendrites and support NR2B delivery [6,48]. Synaptic activity can spatially confine post-ER trafficking in dendrites through CaMK/CaMKII-dependent phosphorylation of KIF17, thereby tuning the length scale of new cargo delivery during plasticity [51].

Mitochondrial dynamics represent an additional critical component of neuronal development, encompassing processes such as fission, fusion, transport, and mitophagy. In developing neurons, mitochondria are strategically positioned in regions of high energy demand, including growth cones, elongating axons, and synapses, to ensure localized ATP production [52]. During neuronal differentiation, cells undergo metabolic reprogramming from glycolysis to oxidative phosphorylation, reflecting increased reliance on mitochondrial function. This transition is accompanied by structural remodeling and redistribution of mitochondria, highlighting the link between metabolism and neuronal maturation [53]. Beyond energy production, mitochondria regulate calcium homeostasis, redox balance, and apoptotic signaling, all of which are essential for neuronal survival and development [52,53]. Their anterograde transport is mediated predominantly by kinesin-1 motors of the KIF5 family, with *KIF5A* playing an important role in neuronal mitochondrial motility through Miro and TRAK adaptor complexes, while dynein supports retrograde transport along microtubules [24,34]. A subset of mitochondria is anchored at specific sites by proteins like syntaphilin, ensuring sustained local energy supply [54]. Disruption of mitochondrial dynamics and transport can impair neuronal development and contribute to neurodevelopmental and neurodegenerative disorders [53].

## 3. Kinesin Gene Families Implicated in Neurodevelopmental Disorders

**Figure 2:**
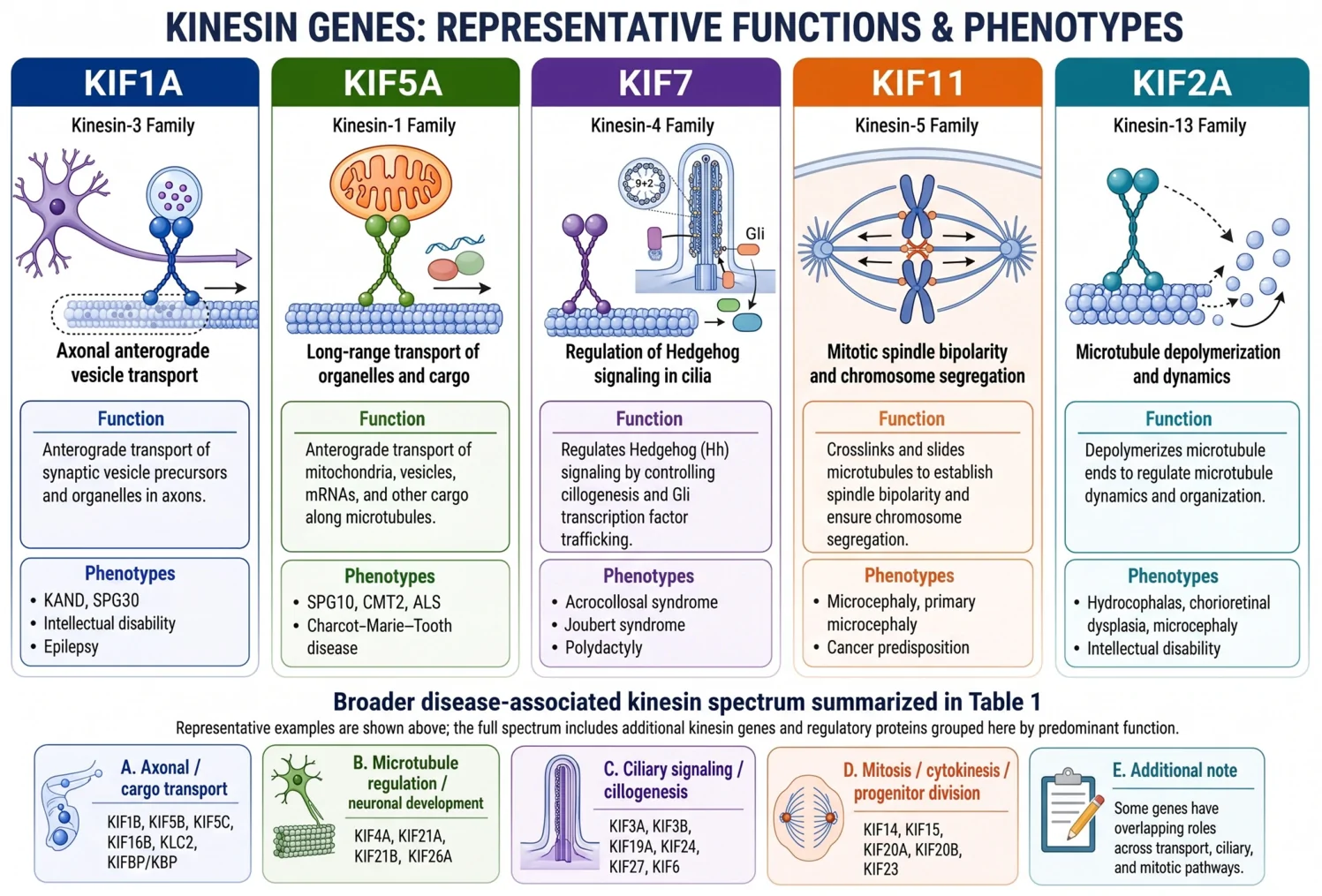
**Representative functions and phenotypes of kinesin genes implicated in neurodevelopmental and neurological disorders.** The upper panels show five representative kinesins, *KIF1A*, *KIF5A*, *KIF7*, *KIF11*, and *KIF2A*, illustrating distinct roles in axonal cargo transport, organelle transport, ciliary Hedgehog signaling, mitotic spindle organization, and microtubule depolymerization. The lower panel places these examples within the broader disease-associated kinesin spectrum by grouping additional genes and regulatory proteins according to their predominant functions in cargo transport, microtubule regulation, ciliary signaling, and mitosis or cytokinesis. Some genes have overlapping roles across these categories.

Kinesins constitute a superfamily of microtubule-based motor proteins comprising approximately 45 genes categorized into 15 subfamilies, each with distinct yet sometimes overlapping roles in intracellular transport, cell division, and developmental processes [7,55]. Although kinesins share a conserved microtubule-binding motor domain, substantial structural diversity exists across the superfamily. Most conventional transport kinesins contain an N-terminal motor domain linked through a stalk region to cargo-binding elements, whereas other families have distinct architectures associated with specialized functions. For example, *KIF2A* is a kinesin-13 microtubule depolymerase rather than a conventional cargo transporter, *KIF11* forms a bipolar tetramer adapted for spindle organization, and KIF26A is an atypical kinesin lacking conventional ATPase-driven motor activity. These structural differences help explain the functional diversity of kinesins in transport, microtubule remodeling, mitosis, and developmental signaling [55]. Increasing evidence highlights the critical contribution of kinesin proteins to vertebrate neurodevelopment, where both functional specialization and genetic compensation between family members influence phenotypic outcomes in disease states [7,56,57,58]. As summarized in Figure 2, the clinical spectrum associated with pathogenic variants in key kinesin family members underscores the tight link between their specialized cellular functions, ranging from axonal transport to mitotic spindle assembly, and the resulting neurodevelopmental phenotypes. 

### 3.1. KIF1A and KIF1A-Associated Neurological Disorders (KANDs)

*KIF1A*, located on chromosome 2q37.3, encodes a neuron-specific ATP-dependent motor protein essential for the axonal transport of synaptic vesicles, organelles, and protein complexes [59]. Pathogenic variants in *KIF1A* give rise to *KIF1A*-associated neurological disorder (KAND), a rare but increasingly recognized neurodegenerative condition characterized by a broad phenotypic spectrum, including hereditary spastic paraplegia (SPG30), intellectual disability, epilepsy, peripheral neuropathy, optic atrophy, and progressive motor dysfunction [60]. In addition to its transport functions, *KIF1A* plays a key role in synaptic transmission and higher-order brain functions such as learning and memory [61]. A hallmark of KAND is marked phenotypic variability, even among individuals harboring identical variants, reflecting both allelic heterogeneity and the influence of variant location within functional domains of the protein [60,62,63]. Motor domain mutations, particularly those affecting ATP binding or microtubule interaction (e.g., p.(Thr99Met) and p.(Glu253Lys)), are associated with severe clinical phenotypes, whereas variants outside these domains often result in milder presentations [62,63,64]. Mechanistically, KAND may arise from haploinsufficiency, dominant-negative effects, or complete loss of functional protein, although the precise molecular consequences, especially for nonsense variants, remain insufficiently characterized [60]. Experimental models further underscore the essential role of *KIF1A*, as knockout mice exhibit early postnatal lethality due to motor and sensory neuron deficits [65]. *KIF1A* variants have also been implicated in a range of neurodevelopmental disorders, including autism spectrum disorder (ASD), Rett syndrome, and hereditary spastic paraplegia, largely through disruption of synaptic vesicle transport and neuronal connectivity [66,67,68,69]. Collectively, these findings establish *KIF1A* as a central regulator of neuronal function and a major contributor to neurodevelopmental disease.

### 3.2. KIF5A and the Spectrum of Axonal Neurodegeneration

*KIF5A*, a member of the kinesin-1 family, is among the earliest identified kinesins and is essential for long-range axonal transport [55]. Pathogenic variants, including missense, frameshift, and *de novo* mutations, have been associated with a broad spectrum of neurodegenerative and neurodevelopmental disorders [10,70,71,72]. Clinically, *KIF5A* variants underlie conditions such as hereditary spastic paraplegia, Charcot-Marie-Tooth disease, amyotrophic lateral sclerosis, and neonatal epileptic encephalopathy [10,11,71,72]. Despite this clinical heterogeneity, several shared molecular mechanisms have been proposed. These include disrupted autoinhibition, altered subcellular localization, mitochondrial transport defects, and the formation of insoluble protein aggregates that impair proteasomal degradation pathways [72]. Additionally, mutant *KIF5A* proteins exhibit reduced stability and turnover, leading to a diminished pool of functional motors available for axonal transport [72]. Domain-specific mutations, particularly those affecting the motor domain, are strongly associated with spastic paraplegia; functional studies show that different *SPG10* variants can reduce microtubule affinity, gliding velocity, or both, while only a subset exhibits a dominant-negative effect at heterozygous ratios [73]. Functional studies in animal models demonstrate that *KIF5A* is indispensable for axonal integrity, as its loss results in abnormal neurofilament transport, neurofilament accumulation, and degeneration of large-caliber axons [74]. KIF21A independently regulates cortical microtubule growth and axon guidance, illustrating how distinct kinesins contribute to neuronal development through complementary mechanisms [75].

### 3.3. KIF7 and Ciliopathy-Related Brain Malformations

*KIF7*, a member of the kinesin-4 family, plays a pivotal role in the regulation of microtubule dynamics and Sonic Hedgehog (Shh) signaling within primary cilia [76,77,78,79]. Through these functions, *KIF7* contributes to key developmental processes, including limb patterning and brain morphogenesis. Variants in *KIF7* disrupt ciliary structure and function, leading to ciliopathies such as Joubert syndrome and acrocallosal syndrome, often characterized by severe brain malformations [20,80,81]. At the cellular level, *KIF7* deficiency results in abnormal ciliary architecture, including elongation, twisting, and instability, which in turn perturb Shh signaling pathways [77]. Genetic studies have identified both sporadic and founder mutations, particularly in specific populations, underscoring the importance of *KIF7* in human disease [81]. Experimental models further demonstrate that *KIF7*-null mice exhibit embryonic lethality, polydactyly, and aberrant neuronal patterning, consistent with its critical developmental role [77]. *KIF7*-associated disease can also include hydrocephalus [82], and clinical and experimental evidence suggests a mechanistic link between *KIF7*- and *C5orf42*-related ciliopathies through Sonic Hedgehog signaling [83].

### 3.4. KIF11 and Mitotic Dysfunction in Neurodevelopment

*KIF11* (kinesin-5) is a tetrameric motor protein essential for bipolar spindle formation and chromosome segregation during mitosis [84,85]. Beyond its canonical role in cell division, *KIF11* also participates in axonal growth, intracellular microtubule transport, and neuronal migration [86], as well as ciliogenesis [87,88]. Pathogenic variants in *KIF11* give rise to a spectrum of syndromic conditions, including microcephaly, chorioretinopathy, lymphedema, and intellectual disability [89,90,91]. These phenotypes are closely linked to mitotic dysfunction, and experimental loss of *KIF11* can cause monopolar spindle formation, chromosome misalignment, chromosome instability, and cell-cycle arrest [92]. In addition, impaired ciliary dynamics and disrupted Hedgehog signaling have been observed following *KIF11* knockdown [87,88]. Reported pathogenic variants include missense changes affecting conserved residues and truncating variants, typically in the heterozygous state [89,90,91,93]. Animal models further confirm the essential role of *KIF11;* homozygous germline loss causes preimplantation lethality, whereas conditional endothelial loss produces severe retinal and milder cerebellar vascular hypoplasia that recapitulates aspects of the human ocular phenotype [94].

### 3.5. KIF2A and Cortical Developmental Disorders

*KIF2A*, belonging to the kinesin-13 family, functions as a microtubule depolymerizing motor critical for neuronal development, migration, and synaptic connectivity [95]. Variants in *KIF2A* are associated with a wide range of cortical malformations, including microcephaly, lissencephaly, pachygyria, epilepsy, and autism spectrum disorder [96,97,98,99,100]. Moreover, rare-variant association analyses have implicated *KIF2A* as a candidate susceptibility locus for Alzheimer’s disease [101]. The pathogenic mechanisms underlying *KIF2A*-related disorders involve disruption of microtubule dynamics, impaired intracellular transport, and defective neuronal polarity and connectivity [95]. Loss of *KIF2A* function results in synaptic loss, axonal degeneration, and altered lysosomal trafficking, contributing to autophagic stress and neurodegeneration [95,102,103]. Notably, *KIF2A* variants may exert dominant-negative or gain-of-function effects, depending on their interaction with cellular partners [95,104]. Animal studies further highlight the critical developmental role of *KIF2A*. *KIF2A*-null mice are born alive but die within one day and show severe brain abnormalities, while conditional or disease-model mutations lead to reduced brain size, impaired neuronal migration, and disrupted cortical organization [105,106,107,108,109,110]. These findings underscore the essential role of *KIF2A* in both embryonic neurogenesis and postnatal neuronal maintenance.

### 3.6. Emerging Roles of KIF14, KIF15, and KIF16B

Recent studies have identified additional kinesins, including *KIF14*, *KIF15*, and *KIF16B*, as contributors or emerging candidates in developmental disease, including phenotypes with microcephaly and intellectual disability. *KIF14* plays a critical role in cytokinesis, and its loss-of-function mutations impair cell division, leading to primary microcephaly with variable severity ranging from fetal lethality to mild developmental delay [111,112,113]. *KIF14* is also implicated in kidney development, with pathogenic variants causing renal developmental abnormalities in some affected individuals [113,114]. Loss-of-function variation in *KIF15* has been reported in a Braddock–Carey syndrome genocopy that included microcephaly, congenital thrombocytopenia, and Pierre–Robin sequence, supporting an emerging human developmental-disease association for this mitotic kinesin [115]. *KIF16B*, a kinesin-3 family member, is involved in intracellular receptor trafficking during early embryogenesis [116]. Variants in *KIF16B* have been linked to intellectual disability, likely through disruption of endosomal transport pathways [117]. These findings support the expanding spectrum of kinesins involved in human neurodevelopment.

### 3.7. Other Kinesins in Neurodevelopment and Disease

A growing body of evidence implicates numerous additional kinesins in neurodevelopmental and neurological disorders. Members of the kinesin-1 and kinesin-2 families, such as KIF5B and KIF3A/B, are essential for embryonic development and ciliogenesis; *Kif5b*-null mice show severe growth retardation and embryonic lethality [118], whereas KIF3A loss disrupts embryonic ciliary morphogenesis [119]. KIF13A and KIF13B exhibit functional redundancy and are collectively required for normal craniofacial development, as combined loss produces perinatal lethality with craniofacial developmental abnormalities [120]; KIF13B also has established roles in receptor trafficking and ciliary signaling [121,122]. KIF21B has emerged as a regulator of neuronal migration and microtubule dynamics, with mutations causing microcephaly through dysregulated motor activity [123]. KIF27 and KIF19A are involved in ciliary function. *Kif27*-deficient mice develop severe congenital hydrocephalus [124], whereas KIF19A regulates ciliary length through microtubule-depolymerizing activity and Kif19a loss can cause hydrocephalus [125,126]. KIF20A and KIF20B regulate neural progenitor division and cortical growth [127,128], while KIF3B mutations have been associated with altered NMDAR trafficking and schizophrenia-like phenotypes in mice [129]. Additional kinesins, including KIF6, KIF24, and KIF26A, contribute to ependymal ciliary function, ciliogenesis/skeletal development, and neural signaling pathways, respectively [18,130,131,132,133].

Importantly, genetic association studies have linked *KIF1B*, *KIF5A*, and *KIF21B* loci to susceptibility to multiple sclerosis, further highlighting the broader neurological relevance of kinesin genes [134,135,136]. *KIF5C*, in particular, plays a critical role in cortical development, with mutations causing pachygyria, epilepsy, and severe neurodevelopmental impairment through disruption of ATP hydrolysis and microtubule binding [97,137,138,139].

## 4. Mechanisms Linking Kinesin Dysfunction to Disease

Kinesin dysfunction converges on a limited set of pathogenic programs that collectively explain much of the cellular and clinical heterogeneity of kinesin-related neurological disease. Across neurodevelopmental and neurodegenerative contexts, defects in motor activity, cargo coupling, and microtubule track integrity disrupt long-range transport, perturb mitosis in neural progenitors, derail ciliary signaling, and trigger axonal stress responses. In the developing nervous system, these failures ultimately translate into circuit malformation, synaptic dysfunction, and impaired neuronal survival [140,141,142].

### 4.1. Defective Cargo Delivery and Synaptic Dysfunction

At the level of intracellular trafficking, the most immediate consequence of kinesin impairment is defective cargo delivery to distal neuronal compartments. Kinesin-1, kinesin-3, and related motors are required for the anterograde transport of synaptic vesicle precursors, active-zone components, receptors, mitochondria, lysosomes, RNA granules, and signaling complexes, and disruption of these pathways compromises both synapse formation and synapse maintenance [1,2,44,143,144]. In spastin-deficient neurons, excess tubulin polyglutamylation weakens KIF5 binding and processivity, reducing delivery of presynaptic vesicles and postsynaptic AMPA receptors and thereby lowering excitatory synapse number and impairing memory [140]. This same principle extends to other cargo classes. Pathogenic *KIF5C* variation impairs axonal mitochondrial transport, dendritic spine maturation, presynaptic release probability, and long-term potentiation, linking a primary transport defect to altered synaptic plasticity and cognition [145]. Disruption of the kinesin-1 adaptor syntabulin similarly diminishes presynaptic cargo transport, synapse density, and synaptic transmission, producing autism-like behaviors, and a human *STB* variant incapable of binding kinesin-1 fails to rescue these phenotypes, underscoring the pathogenic importance of motor-adaptor coupling rather than motor dysfunction alone [144]. *KIF1A*-related disease follows the same logic at the level of synaptic vesicle precursor transport. Reduced *KIF1A* function decreases synaptic vesicle precursor delivery, destabilizes axons, and contributes to neuronal degeneration [65,67], whereas hyperactive *KIF1A* states can produce the opposite imbalance, with pathological accumulation of cargo at axon terminals [16]. Together, these findings indicate that kinesin defects rarely act through a single cargo class. Instead, they simultaneously impoverish or misdirect multiple transport streams whose coordinated delivery is required for neurotransmission and structural synaptic maintenance.

### 4.2. Transport Failure as an Initiator of Stress Signaling and Proteinopathy

A particularly important translational insight is that transport failure itself can initiate degenerative signaling. Loss of kinesin-1 light chain KLC1 causes early axonal transport defects, cytoskeletal disorganization, cargo accumulation, and activation of JNK stress pathways, culminating in tau hyperphosphorylation, aggregation, and axonal degeneration [146]. This establishes a mechanistic bridge between primary trafficking defects and secondary proteinopathy. Similar feed-forward loops are evident in tauopathy and Alzheimer’s disease, where hyperphosphorylated tau destabilizes microtubules and impedes kinesin-1 motility, further reducing axonal transport, aggravating synaptic dysfunction, and amplifying cognitive decline [141,147,148]. Pathological alpha-synuclein can impair axonal transport [149], while C9orf72-derived arginine-rich dipeptide repeats directly associate with axonal transport machinery and impede kinesin- and dynein-driven microtubule-based motility [150]. Although these examples are often discussed in degenerative disease, they are mechanistically informative for neurodevelopmental kinesinopathies because they reveal how persistent transport defects can convert an initial trafficking problem into chronic cellular stress and axonal instability.

### 4.3. Mitochondrial, Lysosomal, and RNA Transport Defects

The cargoes affected by kinesin dysfunction are especially consequential because they include organelles and RNA-containing assemblies that sustain axonal metabolism and local proteostasis. Mitochondrial transport defects recur across developmental and degenerative disease models, with reduced anterograde motility leading to ATP depletion, oxidative stress, calcium dysregulation, and structural axon instability [151,152,153]. *KIF5A* deficiency in human motor neurons selectively diminishes mitochondrial motility and SFPQ-RNA granule transport, reducing neurite complexity and regenerative capacity [34]. Similarly, altered TRAK2-dependent mitochondrial adaptor function causes soma-dominant mitochondrial accumulation, energy failure, and oxidative stress in disease contexts involving abnormal tau species [154]. Lysosomal transport is equally critical. Defects in BORC-dependent kinesin recruitment perturb axonal delivery of lysosome-related vesicles, deplete axonal mRNAs encoding ribosomal and mitochondrial proteins, and precipitate axonal swelling and degeneration [44]. These observations highlight a broader principle in neuronal biology: kinesin dysfunction compromises not only delivery of organelles, but also the local translation and quality-control systems required to maintain distal axons. Because axons are metabolically constrained, such failures disproportionately affect long-projecting neurons and help explain selective neuronal vulnerability in disorders dominated by transport defects.

### 4.4. Mitotic Spindle Dysfunction in Neural Progenitors

Kinesin dysfunction also disrupts brain development at an earlier stage by altering mitotic spindle function in neural progenitors. Kinesin-5/Eg5 (*KIF11*), kinesin-6/KIF23, and kinesin-13/*KIF2A* are integral to spindle pole separation, spindle orientation, chromosome segregation, and cytokinesis, and their dysfunction depletes neural stem and progenitor pools through cell-cycle arrest, apoptosis, or inappropriate lineage commitment [155,156,157]. In cortical progenitors, *KIF23* knockdown perturbs spindle orientation and cytokinesis, induces binucleation, and shifts division modes toward premature neurogenesis and apoptosis, thereby reducing progenitor output and predisposing to microcephaly [156]. *KIF11* dysfunction produces monopolar spindles, chromosome misalignment, cell-cycle arrest, and cell death, a canonical route to syndromic microcephaly and other growth abnormalities [92,155,158]. *KIF2A* dysfunction similarly perturbs microtubule dynamics and has been linked to malformations of cortical development and epilepsy, emphasizing that the same motor family can act in both postmitotic transport and proliferative division programs [104,157]. Notably, these mitotic phenotypes are not merely cell-biological curiosities. By altering symmetric-versus-asymmetric division choices, they reduce the size of the neural progenitor pool, distort cortical lamination, and impair the production and placement of neurons required for normal corticogenesis [127,156,159]. In this context, kinesin dysfunction contributes to microcephaly not only by limiting cell number, but also by mispatterning the developmental programs that specify neuronal identity and cortical architecture.

### 4.5. Disrupted Ciliary Transport and Developmental Signaling

A related but mechanistically distinct route to malformation arises from kinesin-dependent ciliary transport. Primary cilia rely on kinesin-2 motors, especially KIF3A-containing complexes and *KIF7*, to support intraflagellar transport and to localize Hedgehog and, in some contexts, Wnt signaling components [160,161,162]. When this system fails, developmental signaling is misread rather than simply absent. Loss of KIF3A or IFT components abolishes cilia and disrupts Hedgehog signal transduction, causing severe brain malformations including heterotopia, neural dysplasia, and defects in forebrain patterning [162,163]. In cortical organoids, disruption of the ciliary phosphatase INPP5E alters ciliary composition, hyperactivates SHH signaling, and converts dorsal cortical identity toward ventral telencephalic fates, demonstrating direct cilium-dependent control of regional specification [164]. *KIF7* loss further perturbs GLI processing, dorsal cortical layering, and corticofugal and thalamocortical axon development, linking ciliary transport defects to both structural and connectivity phenotypes [21]. These findings clarify that kinesin dysfunction in cilia is not simply a morphogenetic defect; it is a signaling defect that rewrites developmental fate decisions and produces enduring circuit-level consequences.

### 4.6. The Microtubule Track and the Tubulin Code

What unites these apparently disparate processes is a common dependence on the same physical substrate: the microtubule cytoskeleton. Kinesin output is exquisitely sensitive to tubulin post-translational modifications, tubulin isotype composition, and the geometry of the microtubule lattice, such that changes in the “track” can phenocopy or magnify defects in the motor itself [165]. Spastin is a microtubule-severing protein that contributes to microtubule remodeling and thereby influences the tubulin code. Loss of spastin-mediated severing results in longer microtubules with increased tubulin polyglutamylation, a post-translational modification in which glutamate side chains are added to the C-terminal tails of tubulin and modulate interactions with microtubule-associated proteins and molecular motors. In spastin-deficient neurons, this hyper-polyglutamylated state reduces KIF5 binding and processivity, impairing synaptic cargo transport. Notably, experimentally reducing tubulin polyglutamylation restores KIF5-dependent transport and partially rescues excitatory synapse loss, demonstrating a functional link between spastin-mediated microtubule remodeling, the tubulin code, and kinesin activity [140]. Likewise, neuronal TUBB3 levels tune microtubule growth and polyglutamylation, and lowering Tubb3 can increase *KIF5C* motility and enhance delivery of synaptic cargo such as N-cadherin, demonstrating that activity-dependent tuning of tubulin composition can modulate kinesin-based transport [166]. Pathogenic *TUBB3* and *KIF21A* variants add another layer of complexity by disrupting microtubule dynamics and kinesin-microtubule interactions, leading to cranial nerve miswiring, impaired axon guidance, and cortical malformations [141]. These observations are important because they show that disease can emerge from either side of the transport interface: the motor may be defective, the track may be abnormal, or both may be maladapted in a mutually reinforcing way.

### 4.7. Convergence on Shared Cellular Endpoints

The developmental and degenerative consequences of these transport failures therefore converge on a small set of common cellular endpoints. In developing neurons, impaired kinesin-dependent trafficking reduces delivery of synaptic proteins, organelles, and RNAs needed for growth cone advance, axon specification, dendritic maturation, and synaptogenesis, producing abnormal connectivity and circuit assembly. In neural progenitors, defects in mitotic kinesins and ciliary kinesins alter division symmetry and signaling competence, shrinking progenitor pools and distorting cortical patterning. In mature neurons, chronic failure of organelle transport, local translation, and proteostasis drives metabolic collapse, stress signaling, and progressive axonal degeneration. The clinical heterogeneity of kinesin-related disorders thus reflects not a lack of mechanistic coherence but rather the fact that multiple kinesin-dependent processes are deployed at different stages of neural development and in different cellular compartments. A concise mechanistic perspective is that kinesin dysfunction causes disease by disrupting a transport axis that is simultaneously structural, metabolic, and signaling-based: when cargo delivery fails, when progenitors divide improperly, when ciliary signaling is misrouted, or when axons can no longer sustain long-distance trafficking, the nervous system responds with miswiring, malformation, synaptic failure, and degeneration [68,140,141,146,162].

## 5. Model Systems and Experimental Insights

Experimental models have been essential for turning kinesin biology from a collection of candidate mechanisms into a framework for disease causation. Across worms, flies, zebrafish, mice, and human cellular systems, these models have shown that apparently diverse kinesin variants converge on a limited set of cellular failures: impaired long-range transport, defective organelle positioning, altered signaling, and disrupted neuronal maturation. Kinesinopathies are therefore best understood not as isolated motor defects, but as disorders of coordinated transport regulation whose consequences depend on developmental stage, cargo class, and cellular context.

### 5.1. Animal Models of Kinesinopathies

Animal models have been particularly powerful for linking variant class to pathogenic mechanism *in vivo*. In *C. elegans*, knock-in of the patient-equivalent *KIF5B* variant in *unc-116* produces developmental and locomotor abnormalities together with impaired neuronal mitochondrial transport, supporting a dominant-negative transport defect rather than a simple loss of function. The corresponding mouse *Kif5b* model adds a second dimension by suggesting that some alleles may be incompatible with viability, underscoring the developmental essentiality of kinesin-1 family function and the limits of what null or hypomorphic models can capture [167]. Zebrafish studies have been especially informative for developmental phenotypes because they combine transparent embryology with a conserved nervous system architecture; *kif5b* variants disrupt lysosome, autophagosome, mitochondria, and cilium organization, and this multi-organelle trafficking failure mirrors the hypotonia and seizures seen in human disease [168]. Together, these vertebrate systems establish a key principle. Developmental phenotypes often emerge not from one transport lesion, but from the failure of multiple cargo streams that normally act in concert to build and stabilize neurons.

Invertebrate models provide a distinct but equally important experimental window into kinesinopathies. In *Drosophila melanogaster*, the neuromuscular junction (NMJ) enables transport defects to be assessed through synaptic architecture, growth, and maturation. *UNC-104*, the *Drosophila KIF1A* ortholog, has been especially informative for active-zone assembly. Partial loss of *UNC-104* causes site-specific failure of synapse maturation and loss of active-zone components including Brp, Cac, Liprin-alpha, and SRPK79D, showing that kinesin-3 dysfunction can arrest synaptic maturation rather than merely lowering bulk cargo delivery [169]. Complementary *Caenorhabditis elegans* studies have defined the regulatory logic of UNC-104/*KIF1A*. Disruption of UNC-104 autoinhibition can drive excessive or mislocalized presynaptic cargo delivery and alter synapse size and density [170]. ARL-8, an Arf-like small GTPase associated with presynaptic vesicle precursors, promotes their anterograde transport and prevents premature cargo clustering [170,171], while the BLOC-one-related complex (BORC) acts upstream of ARL-8 to support UNC-104-dependent presynaptic cargo movement [172]. Together, these invertebrate studies show that disease-associated transport defects can arise from failure of the motor, failure of cargo organization, or failure of the regulatory logic that couples the two.

Fly models have also been indispensable for distinguishing transport defects from downstream stress signaling. A particularly important insight is that unc-104 dysfunction activates the Wnd/DLK injury pathway, which then constrains presynaptic protein levels and contributes to synaptic failure [173]. This mechanism adds a second layer to kinesin-dependent pathology: transport defects are not merely passive deficits in cargo delivery, but can be interpreted by neurons as damage signals that reprogram synaptic maintenance and remodeling. That distinction is difficult to resolve in mammalian systems, where transport failure, injury signaling, and degeneration often unfold simultaneously, but in *Drosophila* these processes can be separated genetically and temporally. The result is a more complete mechanistic picture in which kinesin dysfunction initiates both direct cargo mislocalization and indirect stress-response cascades that reshape synapse development. Importantly, this logic remains highly relevant to vertebrate disease, where chronic transport failure can similarly feed into progressive synaptic instability and axonal degeneration, even when the initiating lesion is developmental rather than overtly degenerative.

The broader value of *Drosophila* lies in its ability to expose conserved principles of kinesin regulation that are difficult to isolate in vertebrate neurons. Work on kinesin-1 autoinhibition has shown that motor folding is itself a regulatory state, not a passive structural feature [174], and *Drosophila* experiments demonstrate that relieving autoinhibition alters cargo routing, causes axonal mislocalization of dendritic Golgi outposts, depletes kinesin-1 from dendrites, and perturbs the balance with dynein-dependent trafficking [175]. Although flies cannot model human cortical architecture, they have been exceptionally effective for defining how autoinhibition, adaptor engagement, and cargo organization determine whether transport supports growth, polarity, or synaptic maintenance. That is why fly studies remain useful for human kinesinopathies; they reveal generalizable transport logic, including the idea that the same motor can produce different phenotypes depending on whether regulation is lost, cargo selection is altered, or stress signaling is secondarily activated [173,175].

### 5.2. Patient-Derived Cellular Models of Kinesinopathies

Human patient-derived cellular models have extended these insights by placing kinesin variants in a disease-relevant genetic background. Induced pluripotent stem cell-derived neurons and motor neurons now allow direct analysis of intracellular transport, organelle homeostasis, and network maturation in human cells that retain the patient’s full variant constellation and modifying background [176,177,178]. In *KIF1A*-mutant motor neurons, patient-derived cells reproduce neurite fragmentation, neurofilament-positive swellings, proximal *KIF1A* aggregation, lysosomal cargo accumulation, autophagy defects, and increased cell death, providing a human cellular correlate of axonal degeneration and transport stress [179]. *KIF5A*-mutant iPSC motor neurons similarly show reduced anterograde mitochondrial and lysosomal transport, distal *KIF5A* accumulation, and neuritic pathology, while isogenic correction sharpens the causal assignment of these phenotypes to the variant itself [180]. These models are particularly valuable because they reveal not only whether a variant disrupts transport, but also which cargo classes are most sensitive and whether phenotypes emerge during early differentiation, synaptic maturation, or longer-term maintenance. Human iPSC-derived organoids have added a developmental layer to this analysis: KIF26A loss-of-function impairs radial migration, neurite growth, and neuronal survival in organoid models, while also altering MAPK, MYC, and E2F-associated programs, linking a kinesin-like protein to cortical development through both structural and signaling mechanisms [17]. That broader signaling dimension is important because it shows that some kinesin-family proteins act less as canonical motors and more as organizers of developmental state.

Human cellular systems are especially powerful for genotype–phenotype correlation because they preserve patient-specific modifiers that are often lost in engineered animal lines. This makes them ideal for comparing variants that behave similarly in biophysical assays but diverge in clinical severity. iPSC-based systems also offer functional readouts that are directly relevant to disease biology, including neurite length, axonal swellings, mitochondrial dynamics, lysosomal distribution, autophagy, and spontaneous or evoked network activity. Multielectrode recordings have shown that patient-derived neuronal networks can capture disease- and variant-associated changes in bursting, excitability, and synaptic connectivity, thereby bridging cellular defects to circuit-level dysfunction [181,182,183]. In the context of kinesinopathies, this is especially useful because the clinical phenotype often reflects a mismatch between what a motor does in single-molecule assays and what it does within a developing human neuron. Cellular systems therefore provide the missing middle layer between molecular biophysics and clinical presentation.

### 5.3. Isogenic Modeling of Kinesin Dysfunction

CRISPR/Cas9-based approaches have sharpened this translational value by enabling isogenic comparisons and precise mechanism testing. The clearest kinesin-focused example is the knock-in of a patient KIF5B p.(Thr87Ile) variant into *unc-116* in *C. elegans* and into mouse *Kif5b*, which allowed direct assessment of locomotion, body morphology, and neuronal mitochondrial transport *in vivo*. In the worm, heterozygous knock-in animals displayed phenotypes not seen in deletion heterozygotes, and these were suppressed by additional wild-type copies, strongly supporting a dominant-negative mechanism rather than simple haploinsufficiency [167]. Time-lapse mitochondrial imaging further confirmed that the variant impairs axonal transport directly. This illustrates one of the most important uses of genome editing in kinesin research, distinguishing loss-of-function from gain-of-function or dominant-negative effects, which is essential for choosing the correct therapeutic strategy. Broader CRISPR literature, including work in neurological disease models and patient-derived stem cells, shows that the same logic can be extended to generate isogenic controls, introduce or correct human variants, and test whether a phenotype is rescued by restoring the endogenous allele or by modulating a downstream pathway [184,185,186]. For kinesinopathies, that means CRISPR is not only a modeling tool but also a way to define whether a candidate VUS is likely pathogenic, and by what mechanism.

### 5.4. Key Take-Home Messages

Taken together, these model systems show that kinesin dysfunction can be studied at every relevant biological scale. Animal models establish developmental and behavioral consequences; iPSC-derived neurons reveal human-specific cargo, organelle, and synaptic defects; and CRISPR-based engineering links genotype to mechanism with unusual precision. The major translational lesson is that no single model is sufficient on its own. An additional limitation is that no current experimental system fully reproduces the anatomical scale and sustained transport demands of human projection neurons. Human axons can extend over very long distances, approaching 1 m in some projection pathways, whereas commonly used animal and *in vitro* models operate over substantially shorter distances. Consequently, these systems may underestimate transport defects that become particularly important during long-term maintenance of human axons [1]. Zebrafish and mouse models best capture developmental timing and circuit assembly; *Drosophila* excels at revealing conserved logic in cargo selection, autoinhibition, and stress signaling; human iPSC systems capture patient-specific cellular vulnerability, and genome editing allows the same variant to be interpreted across contexts. As these platforms are integrated more tightly, they should accelerate the move from descriptive kinesinopathies toward variant-resolved precision medicine, in which diagnostic interpretation, prognostic counseling, and eventual therapeutic design are all grounded in mechanism rather than gene name alone.

## 6. Translational and Clinical Perspectives

### 6.1. Clinical Heterogeneity

The expanding genetic and mechanistic landscape of kinesin biology is steadily redefining a subset of neurodevelopmental disorders as bona fide kinesinopathies. Pathogenic variation in *KIF1A* [60,62,63,187], *KIF2A* [96,97,98,99], *KIF4A* [188], *KIF5C* [97,137,139,145], *KIF21A* [75,141], *KIF21B* [123], and kinesin regulators such as *KBP*/*KIFBP* [14] is now recognized across a broad clinical spectrum that includes intellectual disability, epilepsy, autism spectrum disorder, microcephaly, ocular motility disorders, axonal neuropathy, and malformations of cortical development. This shift has immediate diagnostic consequences. Kinesin genes are moving from the periphery of sequencing interpretation to the core of neurodevelopmental gene panels, and contemporary reviews support increasingly systematic, mechanism-informed diagnostic interpretation [60,68,157,187]. The value of this approach is amplified when variant interpretation is informed by motor-domain biology, because clinically similar presentations may arise from qualitatively different molecular lesions, including loss of motility, altered microtubule engagement, or relief of autoinhibition with consequent motor hyperactivity [16,75,123,141].

A major conceptual advance is that genotype–phenotype relationships are now becoming detailed enough to be mechanistically interpretable rather than merely descriptive. *KIF1A* studies show that different amino-acid substitutions at the same residue can produce distinct single-molecule motility phenotypes and clinical outcomes, providing a clear example of how motor biophysics can help to predict disease burden in humans [62,63]. Likewise, *KIF21B* missense variants that disrupt autoinhibition cause aberrant motor activation and are associated with microcephaly and corpus callosum agenesis [123], whereas *KIF21A* variations relieve cortical autoinhibition, alter growth-cone behavior, and underlie congenital fibrosis of the extraocular muscles type 1 (CFEOM1) [75]. *KIF4A* variants extend this logic beyond transport itself. KIF4A has established roles in mitotic cell-cycle control [189] and activity-dependent neuronal survival [190], while the kinesin superfamily more broadly also participates in ciliary function and ciliogenesis [76,188]. Clinically, *KIF4A* variants cause X-linked neurodevelopmental disease with intellectual disability, epilepsy, hydrocephalus, and other structural brain abnormalities [188]. These observations make clear that kinesin-related disease cannot be reduced to a simple transport deficit; each mutation class can imprint a distinct molecular phenotype that is then translated into altered neuronal migration, axon specification, branching, and synaptogenesis. At the same time, phenotype assignment remains complicated by marked intragenic heterogeneity, incomplete penetrance, and the persistent challenge of variants of uncertain significance, particularly in large genes with multiple functional domains. Functional validation is therefore becoming as important as sequence detection itself, especially for distinguishing pathogenic motor defects from tolerated variation [68,141,142,168].

Interpretation is further complicated by the fact that kinesin networks are not simply modular but only partially redundant. Evidence from model systems indicates that compensation among family members is highly context-dependent. In zebrafish, loss of a single kif5 isoform causes severe axonal degeneration despite expression of other family members, implying limited rescue capacity *in vivo* [57]. By contrast, cultured-cell studies show that *KIF5A* or *KIF5C* can rescue abnormal mitochondrial distribution in KIF5B-deficient cells, demonstrating that substitution is possible under selected conditions [58]. Even in the intact nervous system, conditional KIF5B loss reveals non-redundant roles in dendritic trafficking, synaptic plasticity, and memory [191]. These findings argue that clinical variability may reflect not only the primary mutation but also the degree to which other motors, adaptors, and regulatory proteins can buffer the defect. That idea is reinforced by the discovery that loss of kinesin-binding protein (KBP; encoded by *KIFBP*) perturbs microtubule access of specific kinesins, including *KIF1A* and KIF18A, contributing to Goldberg–Shprintzen syndrome and related developmental phenotypes [14]. Collectively, these studies suggest that motor compensation is real, but incomplete, and that its extent may itself be an important determinant of clinical expressivity.

### 6.2. Precision Medicine Approaches

**Figure 3:**
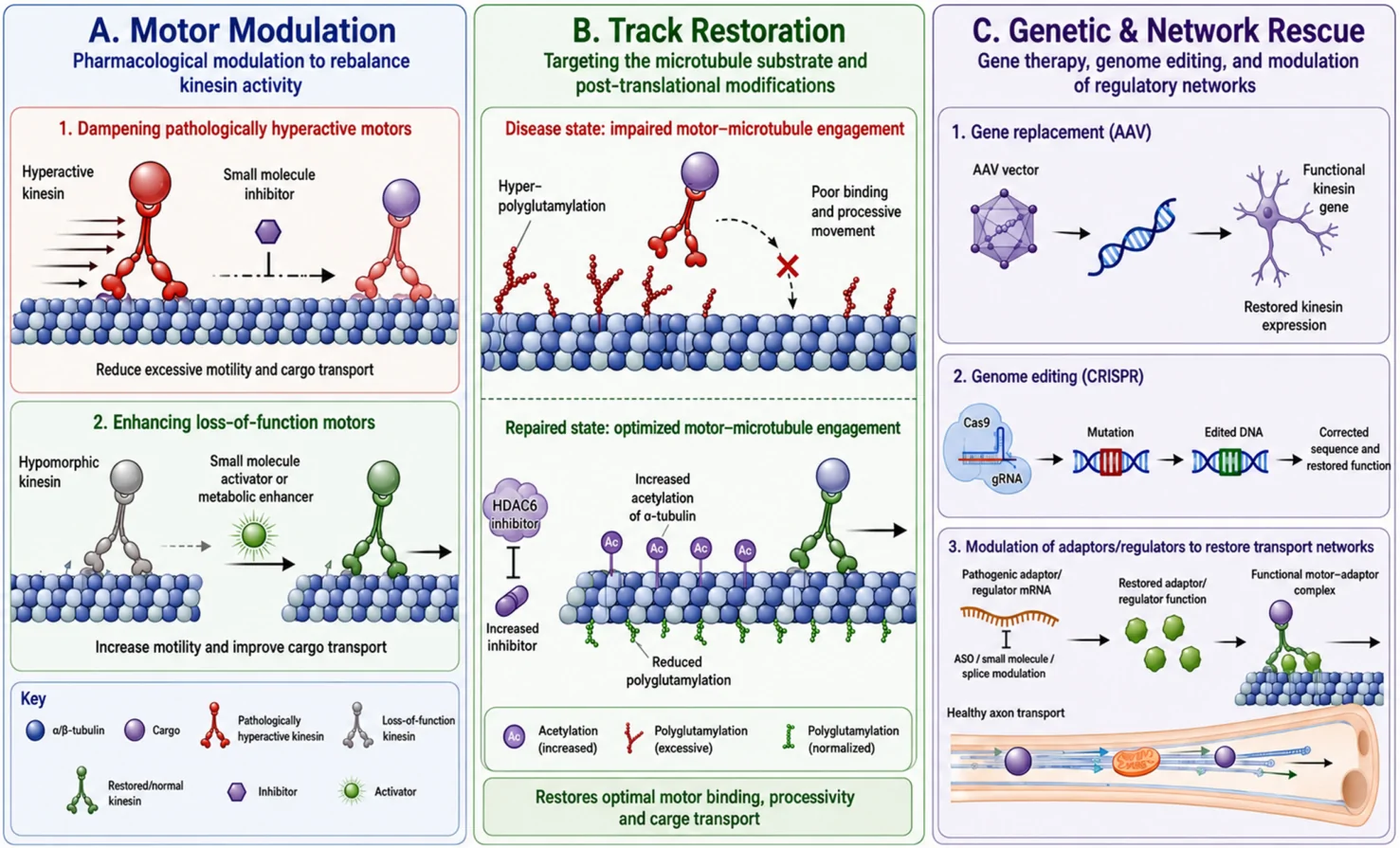
**Emerging therapeutic strategies for kinesin-associated neurodevelopmental disorders.** (**A**) Pharmacological motor modulation aims to rebalance kinesin activity by inhibiting pathologically hyperactive motors or enhancing residual function in loss-of-function states. (**B**) Track-directed approaches target the microtubule substrate and its post-translational modifications, including increased α-tubulin acetylation and normalization of excessive polyglutamylation, to improve motor–microtubule engagement, processivity, and cargo transport. (**C**) Gene- and network-based strategies include AAV-mediated gene replacement, CRISPR-based genome editing, and modulation of pathogenic adaptor or regulatory transcripts through antisense oligonucleotides, small molecules, or splice-modifying approaches to restore functional motor–adaptor complexes and axonal transport. Together, these complementary strategies seek to rescue neuronal trafficking defects and improve neurodevelopmental outcomes in kinesinopathies, although most remain at the preclinical stage.

These mechanistic insights are beginning to shape precision-medicine thinking, although translation remains early and largely preclinical. The observation that specific *KIF1A* variants produce distinct motility abnormalities has encouraged the idea of variant-tailored intervention strategies, in which therapy would be matched to the underlying biophysical defect rather than the gene name alone [68]. For *KIF5B*-related disease, patient-derived studies showing that reduced mTOR signaling can be partially rescued by leucine supplementation provide proof-of-principle that downstream pathway modulation may be therapeutically informative when the motor defect is not directly druggable [167]. More generally, current work points to three therapeutic axes: rebalancing motor activity, repairing the microtubule substrate, and restoring cargo flow (Figure 3).

Pharmacological modulation of kinesin function illustrates both the promise and the biological constraints of this strategy. In tauopathy models, partial suppression of kinesin-1 activity reduces tau hyperphosphorylation, aggregation, and memory impairment, indicating that inhibiting transport can be beneficial when transport becomes pathologically redirected or overloaded [148]. Yet that same principle would likely be harmful in developmental kinesinopathies where baseline transport is already insufficient, underscoring the need for mutation-specific rather than class-wide treatment rules. Conversely, there are contexts in which increasing transport is advantageous. KIF9 overexpression enhances lysosomal trafficking, augments macroautophagy, and reduces amyloid burden and cognitive dysfunction in an Alzheimer disease mouse model, highlighting kinesin-driven clearance pathways as a tractable therapeutic entry point [192]. Reviews therefore increasingly advocate the idea of fine-tuned kinesin modulation, either inhibition or restoration, depending on whether the mutation produces hyperactivity, mislocalization, or loss of function [75,123,142]. The therapeutic logic is thus shifting away from “more transport is better” toward “the right transport, in the right place, at the right time.”

A parallel line of work targets the microtubule track itself, and this may ultimately prove more generalizable than direct motor manipulation. Disease-associated changes in the tubulin code, particularly hyper-polyglutamylation, weaken kinesin binding and impair processivity [140,193]. In spastin-deficient neurons, lowering polyglutamylation restores KIF5 transport and partially rescues excitatory synapse loss, providing a direct demonstration that correcting microtubule chemistry can normalize motor performance [140]. The newer literature extends this principle in two important directions. First, increasing α-tubulin K40 acetylation promotes kinesin-1 binding and improves cargo delivery to neurite tips, suggesting that acetylated microtubules can act as preferred tracks for selected transport programs [194]. Second, HDAC6 inhibition increases tubulin acetylation and rescues BDNF vesicle transport and activity-dependent release, reinforcing acetylation as a therapeutically relevant state in disorders with transport failure [195]. In addition, activity-dependent reduction in Tubb3 alters microtubule posttranslational modifications, increases *KIF5C* motility, and enhances synaptic cargo transport, indicating that tubulin isotype composition itself can be leveraged to tune kinesin-based trafficking [166]. These studies collectively argue that the microtubule cytoskeleton is not a passive scaffold but a dynamic regulatory platform whose biochemical state can determine whether transport succeeds or fails. They also caution, however, that the same interventions are unlikely to be universally beneficial, because the optimal microtubule state may differ across developmental stage, neuronal subtype, and motor–cargo pair.

Gene-based therapy offers the most direct route to mechanistic correction, but also faces the steepest translational barriers. Adeno-associated virus (AAV)-mediated gene replacement, CRISPR-based repair, and variant-specific genome editing are conceptually well suited to monogenic neurodevelopmental disorders, although their application to kinesinopathies remains largely preclinical [184,185,186,196]. This is especially relevant for *KIF21A*, *KIF21B*, and certain *KIF1A* variants, where pathogenesis reflects inappropriate motor activation or altered regulation rather than simple absence of protein [16,68,75,123]. The therapeutic relevance of endogenous regulators should not be overlooked either; KBP is itself a nodal controller of kinesin access to microtubules and therefore a potential intervention point in syndromic neurodevelopmental disease [14]. In parallel, adaptor proteins such as JIP3 are emerging as candidate targets for antisense-based modulation, supported by experimental antisense-oligonucleotide reduction in JIP3 in the context of a toxic gain-of-function *MAPK8IP3* variant [197]. Yet all of these strategies must confront a shared set of obstacles: broad neuronal distribution, developmental timing, dose sensitivity, and the risk of perturbing a transport network that is already delicately balanced. These concerns are particularly acute for microtubule-targeting agents, whose systemic toxicities in oncology underscore how difficult it is to modulate cytoskeletal dynamics safely and selectively in the nervous system [198,199].

### 6.3. Challenges and Pitfalls

Several unresolved issues now define the frontier of the field. The first is the scope of compensation among kinesin family members: the literature clearly shows that redundancy exists, but only in certain contexts, and it remains unclear which cargos, compartments, or developmental windows are permissive to substitution [57,58,191]. The second is selective neuronal vulnerability, which likely reflects a combination of motor specificity, cargo dependence, developmental timing, and regional differences in microtubule architecture, yet remains poorly understood. The third is the interaction between kinesins and the microtubule environment, including tubulin isotypes, posttranslational modifications, and microtubule-associated proteins such as tau, all of which can reshape transport without altering the motor itself [141,147,165]. Structural elements within the kinesin motor domain can also critically tune microtubule engagement; for example, a conserved 3(10) helix is required for normal kinesin function and is relevant to human disease [200]. Finally, network adaptation over time remains an open question. Compensatory changes may initially preserve transport but could also create new imbalances, complicating straightforward therapeutic augmentation. Recent post-translational-modification studies sharpen this point by suggesting that adaptation may occur at the level of the track as well as the motor, through altered acetylation, glutamylation, and tubulin composition [140,166,194].

Looking ahead, the most credible therapeutic opportunities will probably emerge from a convergence of approaches rather than from any single intervention class. Variant-resolved functional assays, patient-derived neuronal models, and *in vivo* systems will be needed to connect genotype to motor behavior and then to circuit-level pathology. The most promising strategies are likely to be those that either restore a clearly defined loss-of-function transport defect or selectively dampen a hyperactive, misregulated motor, while microtubule-directed therapies may offer a broader but more technically demanding route to correcting transport failure. The major unanswered questions remain the same but are now more sharply framed: how compensation is organized, why certain neurons are uniquely vulnerable, and which transport states can be safely and durably normalized in the developing human brain.

## 7. Future Directions

The next phase of kinesinopathy research will likely be defined less by the discovery of additional disease genes than by a deeper understanding of how kinesin-dependent transport is organized across developmental time, cell type, and subcellular compartment. The central challenge is no longer simply to show that kinesin dysfunction causes neurodevelopmental disease, but to determine how specific defects in motor regulation, cargo selection, and microtubule engagement are translated into distinct cellular and clinical phenotypes. In that sense, the field is moving from gene-centric description toward a transport-centric systems biology of neuronal development [68,140,141,165].

### 7.1. System-Level Data Integration

A major priority will be the integration of genomics with proteomics, transcriptomics, and spatially resolved biology to map kinesin interactomes in the developing and mature brain. Kinesins do not act in isolation; they operate within cargo-specific and cell type-specific transport networks that are shaped by adaptor proteins, track state, local signaling, and developmental context [14,144,165]. Multi-omic approaches should therefore be used to define which cargos are preferentially coupled to particular kinesins in defined neuronal populations, how these interactions change during neuronal migration, axon specification, synaptogenesis, and maturation, and how disease-associated variants rewire these networks [14,144,165]. Such studies will be especially informative when coupled to spatial transcriptomic and proteomic profiling, which can reveal whether a given kinesinopathy reflects failure of a broadly distributed transport program or selective collapse of a vulnerable subcellular domain such as the axon initial segment (AIS), distal axon, dendritic branch, or ciliary compartment [21,160,161,162,163,164]. The most useful future datasets will not merely catalog binding partners, but will connect molecular occupancy to transport behavior and disease trajectory.

This systems-level perspective will also be essential for resolving a growing appreciation that kinesins serve functions beyond classical cargo transport. The emerging literature already suggests that kinesin-dependent trafficking can organize RNA granules and local translation, rather than acting only to move conventional vesicular cargo [5,43,44,201]. That conceptual shift is likely to prove particularly important for neurodevelopment, where transport and fate specification are tightly interwoven. Kinesins may help position RNA and translation machinery within neurites, thereby shaping local proteome composition during growth and synapse assembly [5,43,44,201]. Kinesin-family proteins may also regulate developmental signaling pathways directly or indirectly, as illustrated by KIF26A-dependent GDNF-Ret/MAPK signaling and ciliary kinesin regulation of SHH pathways [17,19,20,21,76,77,81,83]. Future work will need to determine whether these roles reflect cargo transport in a broader sense or whether some kinesin-family proteins function as organizers of intracellular signaling hubs that specify neuronal state. This distinction matters because it may explain why some pathogenic variants produce developmental malformations even when bulk motor activity appears relatively preserved [17,18,19,20,76,77,81,83].

Another unresolved question is how transport regulation is integrated across the different layers emphasized throughout this review, including autoinhibition, adaptor control, cargo identity, microtubule chemistry, and stress signaling. The available evidence argues that kinesinopathy arises when this coordination fails, not simply when a motor is weakened [12,15,16,44,140,144,145,165]. Yet the field still lacks a unified framework for understanding how altered autoinhibition, aberrant cargo coupling, and track defects are combined within individual neurons to produce selective vulnerability. This issue is especially pressing in light of invertebrate studies that have shown how UNC-104/*KIF1A* regulation, cargo organization, synapse maturation, and Wnd/DLK injury signaling can be genetically separated [169,170,171,172,173]. Those findings suggest that chronic transport failure may trigger a secondary stress program that amplifies synaptic or axonal pathology, a principle that likely extends to vertebrate neurons as well. In mammalian systems, however, this hierarchy is harder to disentangle because transport collapse, proteostatic stress, and structural degeneration often coexist [44,141,146,147,148,149,150,168]. Future experimental strategies will therefore need to leverage model systems in which these phases can be temporally and mechanistically resolved.

### 7.2. Shifting the Clinical Paradigms

Longitudinal patient studies will be equally important for translating mechanism into clinically useful stratification. Kinesinopathies span developmental encephalopathy, peripheral neuropathy, spastic paraplegia, epilepsy, optic atrophy, and progressive axonal degeneration, but the temporal relationship between molecular defect and clinical evolution remains poorly defined for many variants [10,60,62,63,71,72,73,96,97,98,99,123,134,135,136,137,139,145,187,188]. Serial studies that combine imaging, electrophysiology, and molecular profiling could identify when transport defects first become measurable, which biomarkers best reflect disease burden, and which phenotypes are stable versus progressive over time [16,34,60,62,68,145,179,180]. Such work will be essential for distinguishing congenital developmental failure from later transport-dependent degeneration, a distinction with direct implications for intervention timing. It will also clarify how genotype–phenotype relationships evolve across developmental stages, especially in disorders where early malformation and later neurodegeneration overlap [60,63,72,95,96,97,98,99,187].

Human cellular models and CRISPR-based engineering will remain central to this effort, but their greatest value will come when they are embedded within broader longitudinal and *in vivo* frameworks. Patient-derived neurons already make it possible to quantify cargo-specific transport defects, mitochondrial dysfunction, synaptic abnormalities, and rescue responses in genetically matched backgrounds. Genome editing extends this by enabling isogenic correction, VUS validation, and mechanistic separation of loss-of-function from dominant-negative or gain-of-function behavior [180,183,184,185,186]. The next step is to align these cellular phenotypes with clinical trajectories and to ask which molecular readouts are most predictive of neurological outcome. That integration will be particularly important for therapeutic development, because kinesinopathies are likely to require individualized strategies: restoring transport where it is deficient, dampening motors that are pathologically hyperactive, stabilizing the microtubule substrate when the track is altered, or modulating downstream stress pathways when transport failure has already initiated a feed-forward injury response [15,16,140,146,150,166,194].

The broader conceptual shift is that kinesin biology is beginning to redefine how neurodevelopmental disease is classified. What initially appeared to be a collection of rare syndromes is increasingly emerging as a set of mechanistically stratified transport disorders, in which the critical variables are not only the gene and variant, but the affected cargo, the developmental window, the vulnerable neuronal compartment, and the capacity for network compensation [14,16,21,44,57,58,68,123,140,145,155]. As multi-omic mapping, improved patient phenotyping, and variant-resolved functional assays converge, kinesinopathies should become increasingly tractable as precision diseases. That will not only sharpen diagnosis and prognosis, but also transform transport biology from a descriptive framework into a basis for rational therapeutic stratification in the developing human brain [14,16,21,44,68,123,140,145,155]. A comprehensive gene-by-gene synthesis of established, emerging, and model-supported kinesin associations, including their principal functions, pathogenic mechanisms, and neurodevelopmental or clinical phenotypes, is provided in Table 1.

## 8. Conclusions

Kinesin dysfunction emerges from this review as a systems-level disorder of neuronal transport rather than a collection of isolated motor defects. Across developmental, synaptic, axonal, and ciliary contexts, disease arises when the coordinated logic of cargo selection, motor regulation, and microtubule engagement fails, jointly tuned to developmental stage and neuronal compartment. This framework explains why clinically overlapping disorders can stem from distinct molecular lesions, why neuronal vulnerability is selective, and why some variants cause malformation whereas others drive degeneration, and it accommodates the growing recognition that kinesins act beyond cargo movement to organize RNA localization, local translation, and signaling hubs. The translational implication is that mechanistic and model-based studies now provide a basis for stratifying kinesinopathies by transport mechanism, vulnerable cargo, and disease stage, enabling precision strategies that restore deficient transport, dampen pathological hyperactivity, stabilize the microtubule substrate, or interrupt secondary stress cascades before irreversible circuit failure. In this sense, kinesin biology is reshaping neurodevelopmental disease from a gene-centered taxonomy into a mechanistically stratified map of neuronal transport vulnerability.

## Figures and Tables

**Table 1:** **Kinesin superfamily genes and regulators implicated in human neurodevelopmental and neurological disorders or supported by neurodevelopmentally relevant experimental models.** Genes are ordered from principal disease-associated kinesins through emerging, candidate, and model-supported members. References are selected to support the principal function, mechanism, and disease/model association summarized in each row and are not intended to be exhaustive.

Gene	Kinesin Family	Principal Molecular Function	Principal Pathogenic Mechanism(s)	Human Disorder(s)/Model Phenotype(s)	Key Neurodevelopmental/Clinical Features	Ref(s)
***KIF1A***	Kinesin-3	Neuron-specific anterograde transport of synaptic vesicle precursors, organelles and protein complexes; synaptic transmission, learning and memory	Loss of motility/processivity (e.g., p.R169T); haploinsufficiency; dominant-negative effects; relief of autoinhibition causing motor hyperactivity	*KIF1A*-associated neurological disorder (KAND); spastic paraplegia (SPG30); NESCAV syndrome	Intellectual disability, epilepsy, spasticity, axonal/peripheral neuropathy, optic atrophy, ASD, Rett-like features; severity influenced by variant position and motor behavior	[15,16,59,60,62,63,64,65,66,67,68,69,187]
***KIF5A***	Kinesin-1 (heavy chain)	Long-range anterograde axonal transport of mitochondria, SFPQ–RNA granules and vesicles	Disrupted autoinhibition/localization; impaired microtubule binding and motility; altered mitochondrial and SFPQ–RNA transport; reduced motor stability/turnover; protein aggregation; domain-dependent effects	Hereditary spastic paraplegia (SPG10); Charcot–Marie–Tooth disease type 2; amyotrophic lateral sclerosis (ALS25); neonatal epileptic encephalopathy	Spasticity, axonal neuropathy, motor-neuron degeneration, neonatal myoclonic seizures and progressive leukoencephalopathy	[10,11,34,70,71,72,73,74,202]
***KIF7***	Kinesin-4	Regulation of microtubule dynamics and Sonic Hedgehog (SHH) signaling at the primary cilium; GLI processing; limb and brain morphogenesis	Disrupted ciliary architecture (elongation, twisting, instability); perturbed SHH/GLI signaling	Acrocallosal syndrome; Joubert syndrome; hydrolethalus syndrome; hydrocephalus in some affected individuals	Corpus callosum anomalies, polydactyly, cortical malformation and corticofugal/thalamocortical connectivity defects	[20,21,76,77,78,79,80,81,82,83]
***KIF11*** *(Eg5)*	Kinesin-5	Bipolar mitotic spindle assembly and chromosome segregation; also neuronal migration/axon growth and ciliogenesis	Mitotic spindle dysfunction (monopolar spindle, chromosome misalignment/instability, cell-cycle arrest); impaired ciliary/Hedgehog dynamics	Microcephaly with or without chorioretinopathy, lymphedema and intellectual disability (MCLMR); familial exudative vitreoretinopathy	Microcephaly, chorioretinopathy, lymphedema, intellectual disability	[84,85,86,87,88,89,90,91,92,93,94,158]
***KIF2A***	Kinesin-13	Microtubule depolymerization; neuronal migration, polarity and collateral-branch suppression; cilium disassembly and spindle dynamics	Disrupted microtubule dynamics; impaired migration/connectivity; dominant-negative or gain-of-function effects; ciliogenesis and cell-cycle defects	Malformations of cortical development (CDCBM3); lissencephaly/pachygyria; microcephaly	Lissencephaly, pachygyria, microcephaly, epilepsy/infantile spasms, intellectual disability, ASD	[9,95,96,97,98,99,100,104,105,106,107,108,109,110,157]
***KIF5C***	Kinesin-1 (heavy chain)	Anterograde axonal and mitochondrial transport; dendritic spine maturation, presynaptic release and LTP	Impaired ATP hydrolysis/microtubule binding; defective mitochondrial transport and synaptic maturation	Malformations of cortical development (CDCBM2)	Pachygyria, epilepsy, severe neurodevelopmental impairment	[58,97,137,138,139,145]
***KIF14***	Kinesin-3	Cytokinesis; also kidney development	Loss-of-function impairing cell division	Primary microcephaly/intellectual disability with microcephaly; renal developmental abnormalities	Microcephaly ranging from fetal lethality to milder developmental delay; variable renal abnormalities	[111,112,113,114]
***KIF15***	Kinesin-12	Mitotic spindle assembly and chromosome segregation (partly redundant with *KIF11*)	Biallelic loss-of-function; impaired mitotic kinesin function (emerging human evidence)	Braddock–Carey syndrome genocopy (single-family report)	Microcephaly, congenital thrombocytopenia, Pierre Robin sequence, corpus callosum agenesis	[115]
***KIF16B***	Kinesin-3	Rab14-dependent endosomal/FGFR trafficking during early embryogenesis	Disrupted endosomal receptor transport	Candidate autosomal-recessive intellectual disability syndrome	Intellectual disability/developmental delay	[116,117]
***KIF26A***	Non-motor/atypical (lacks ATPase)	Negative regulator of GDNF–Ret and MAPK signaling in migrating neurons; radial migration and axon growth	Biallelic loss-of-function dysregulating signaling, impairing migration and increasing apoptosis (not active-transport failure)	Cortical dysplasia, complex, with other brain malformations 11 (CDCBM11); pediatric intestinal pseudo-obstruction (PIPO)	Cortical malformation, developmental delay, enteric/GI dysmotility	[17,18,19]
***KIF21A***	Kinesin-4	Cortical microtubule growth inhibitor; axon guidance	Relief of autoinhibition causing aberrant motor activation; altered growth-cone behavior	Congenital fibrosis of the extraocular muscles type 1 (CFEOM1)	Ocular motility disorder, cranial-nerve miswiring	[75,141]
***KIF21B***	Kinesin-4	Neuronal migration; regulation of microtubule dynamics	Missense variants disrupting autoinhibition, causing aberrant motor activation	Microcephaly with corpus callosum agenesis	Microcephaly, corpus callosum agenesis, intellectual disability	[123]
***KIF4A***	Kinesin-4	Chromosome organization, mitotic progression and cytokinesis; activity-dependent neuronal survival	Disrupted chromokinesin/cell-cycle function; impaired neuronal survival	KIF4A-associated X-linked neurodevelopmental disorder	Developmental delay/intellectual disability, epilepsy, microcephaly, hydrocephalus and variable brain malformations	[188,189,190]
***KIF1B***	Kinesin-3	Anterograde transport of mitochondria, IGF1R and neuronal cargoes	Impaired cargo transport and axon growth; reported susceptibility association	Reported hereditary axonal neuropathy/CMT2A1; multiple sclerosis susceptibility	Peripheral neuropathy and axon-growth defects; reported MS susceptibility	[134,203]
***KIF5B***	Kinesin-1 (heavy chain)	Ubiquitous kinesin-1 transport; mitochondrial, lysosomal and autophagosomal organization; ciliary homeostasis	Dominantly acting transport defects; cargo/organelle mislocalization; null alleles are embryonic-lethal in mice	KIF5B-related pleiotropic disorder, including neurodevelopmental and skeletal phenotypes	Severe hypotonia with or without seizures, developmental delay/ID; variable skeletal, muscular and cardiac involvement	[118,167,168,191]
***KIF3A/KIF3B***	Kinesin-2	Intraflagellar transport; ciliogenesis; Hedgehog signaling	Loss of ciliary transport/signaling; altered NMDAR trafficking in Kif3b mutant mice	Ciliary developmental abnormalities (KIF3A model); schizophrenia-like phenotype (Kif3b model)	Ciliary morphogenesis/forebrain-patterning defects; altered NMDAR trafficking and neurobehavioral phenotype in models	[119,129,160,161,162,163]
***KIF13A/KIF13B***	Kinesin-3	Endosomal/receptor trafficking; functionally redundant	Combined loss-of-function	Combined-loss craniofacial developmental phenotype (model-supported)	Craniofacial abnormalities and perinatal lethality in combined-loss models	[120,121,122]
***KIF23***	Kinesin-6	Central-spindle organization and cytokinesis; spindle orientation in progenitors	Disrupted spindle orientation/cytokinesis causing binucleation, premature neurogenesis and apoptosis	Reduced cortical progenitor pool/microcephaly-like phenotype (model-supported)	Spindle-orientation and cytokinesis defects, binucleation, premature neurogenesis and apoptosis	[156]
***KIF20A/KIF20B***	Kinesin-6	Neural progenitor division and cytokinesis	Impaired progenitor division	Reduced cortical growth/microcephaly-like phenotype (model-supported)	Impaired neural progenitor division, reduced progenitor pool and cortical size	[127,128]
***KLC2***	Kinesin-1 light chain	Cargo coupling within the kinesin-1 complex	Gain-of-function via overexpression (non-coding deletion), destabilizing complex balance	SPOAN syndrome (spastic paraplegia, optic atrophy, neuropathy)	Spastic paraplegia, optic atrophy, peripheral neuropathy	[22]
***KIFBP (KBP;*** **formerly *KIAA1279)***	Kinesin-binding regulator	Controls microtubule access of specific kinesins (e.g., *KIF1A*, KIF18A)	Loss-of-function or reduced KIFBP expression, perturbing kinesin–microtubule access and neuronal development	Goldberg–Shprintzen syndrome	Microcephaly, intellectual disability, peripheral neuropathy, Hirschsprung disease	[14,204]
***KIF27/KIF19A***	Kinesin-4/Kinesin-8	Motile-cilia integrity (KIF27); ciliary length control via microtubule depolymerization (KIF19A)	Disrupted ciliary structure/function	Congenital hydrocephalus (model-supported)	Hydrocephalus and abnormal ciliary structure/length in knockout models	[124,125,126]

***Abbreviations:** ALS, amyotrophic lateral sclerosis; ASD, autism spectrum disorder; ATP, adenosine triphosphate; CDCBM, cortical dysplasia, complex, with other brain malformations; CFEOM1, congenital fibrosis of the extraocular muscles type 1; GI, gastrointestinal; KAND, KIF1A-associated neurological disorder; LTP, long-term potentiation; MCLMR, microcephaly with or without chorioretinopathy, lymphedema, or mental retardation; MS, multiple sclerosis; NESCAV, neurodegeneration and spasticity with or without cerebellar atrophy or cortical visual impairment; PIPO, pediatric intestinal pseudo-obstruction; SHH, Sonic Hedgehog; SPG, spastic paraplegia; SPOAN, spastic paraplegia, optic atrophy and neuropathy. For entries supported primarily by experimental models rather than established human gene–disease associations, the model-supported status is stated explicitly in the table.*

## Data Availability

No new data were created or analyzed in this study. Data sharing is not applicable to this article.
